# Thoracoscopic esophagectomy with subcarinal lymph node dissection in the prone position for esophageal cancer with a right superior pulmonary vein anomaly: a case report

**DOI:** 10.1186/s40792-019-0567-z

**Published:** 2019-01-14

**Authors:** Yu Onodera, Yusuke Taniyama, Tadashi Sakurai, Makoto Hikage, Chiaki Sato, Kai Takaya, Hiroshi Okamoto, Shota Maruyama, Takuro Konno, Michiaki Unno, Takashi Kamei

**Affiliations:** 1 Department of Surgery, Tohoku University Graduate School of Medicine, 1-1 Seiryo-machi, Aoba-ku, Sendai, Miyagi 980-8574 Japan; 2 Department of Surgery, Kesennuma City Hospital, 8-2 Akaiwasuginosawa, Kesennuma, Miyagi 988-0181 Japan; 3 Department of Surgery, Hachinohe City Hospital, 3-1-1 Tamukai, Hachinohe, Aomori 031-8555 Japan

**Keywords:** Thoracoscopic esophagectomy, Thoracoscopy, Prone, Anomaly, Pulmonary vein, Subcarina, Dissection, Virtual thoracoscopy

## Abstract

**Background:**

Superior posterior pulmonary vein anomaly in the right upper lobe (anomalous V2), which is one of the anomalies of the right superior pulmonary vein (RSPV), runs behind the right main or intermediate bronchus. Although this rarely occurs, attention should be given to this venous anomaly during thoracoscopic esophagectomy with subcarinal lymph node dissection. Here, we report a case of thoracoscopic esophagectomy with subcarinal lymph node dissection in the prone position for lower thoracic esophageal cancer with anomaly of the superior posterior pulmonary vein in the right lobe (anomalous V2).

**Case presentation:**

A 61-year-old man was diagnosed as having lower esophageal cancer with swelling of multiple lymph nodes in the mediastinum and abdomen. His clinical diagnosis based on the eighth TNM classification system was cT3 N2 M0 stage IIIB. In addition, an anomalous V2 was recognized on preoperative computed tomography imaging before the operation. The vein ran behind the intermediate bronchus and drained into the RSPV located at the area of the subcarinal lymph node. We performed preoperative simulation by using virtual thoracoscopic imaging with the same view as that during operation to help us better dissect the lymph nodes. As a result, thoracoscopic esophagectomy and subcarinal lymph node dissection were performed in the prone position without injuring the anomalous V2. Severe complications did not occur in the postoperative course except for paralysis of the left recurrent laryngeal nerve. The patient was discharged on postoperative day 17.

**Conclusions:**

Injury to an anomalous V2 can cause severe hemorrhage during subcarinal lymph node dissection in esophagectomy. Preoperative simulation by using virtual thoracoscopic imaging is useful to avoid this complication in patients with an anatomical anomaly.

## Background

Subcarinal lymph node dissection is the standard operative strategy for thoracic esophageal cancer [1–3]. Though rare, right superior pulmonary vein (RSPV) anomaly runs behind the right main or intermediate bronchus [4–6]. Only a few reports have described esophagectomy with subcarinal lymph node dissection for patients with esophageal cancer who had an RSPV anomaly [7–9]. These patients had a successful subcarinal lymph node dissection without injury to the anomalous RSPV, which was not recognized before the operation. Although lymph node dissection might be technically possible without recognizing the RSPV anomaly preoperatively, injury to this vein would cause severe hemorrhage and be fatal. Thus, the preoperative recognition of an RSPV anomaly reduces the risk of injury during operation. Here, we report a case of thoracoscopic esophagectomy with subcarinal lymph node dissection in the prone position for lower thoracic esophageal cancer with anomaly of the superior posterior pulmonary vein in the right lobe (anomalous V2), which is one of the anomalies of the RSPV, in which the anomalous vein was identified before operation on computed tomography (CT).

## Case presentation

**Fig. 1:**
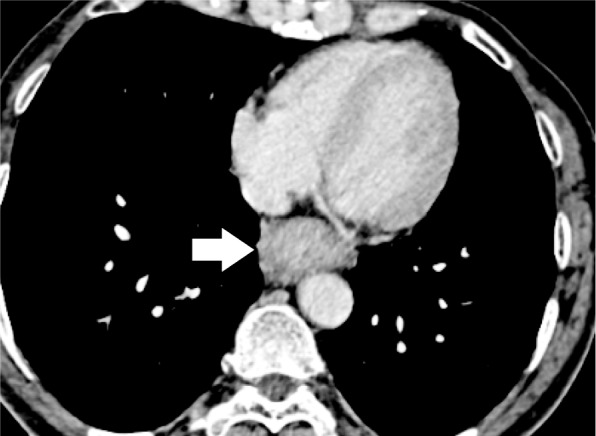
CT image before treatment. Wall thickness of the lower thoracic esophagus (white arrow)

**Fig. 2:**
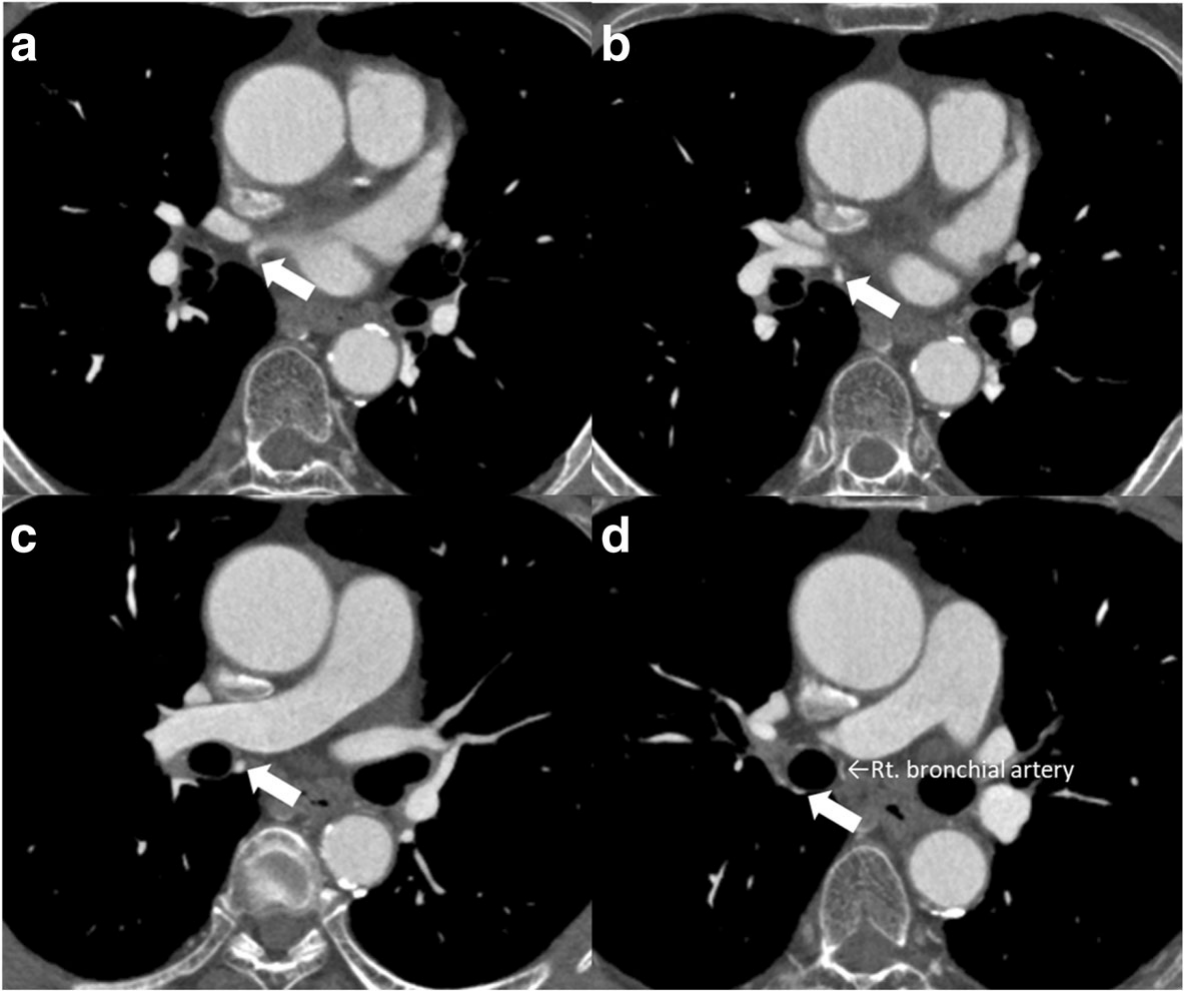
CT image of the course of the anomalous V2. **a** Anomalous V2 draining into the RSPV. **b–d** Anomalous V2 running behind the intermediate bronchus

**Fig. 3:**
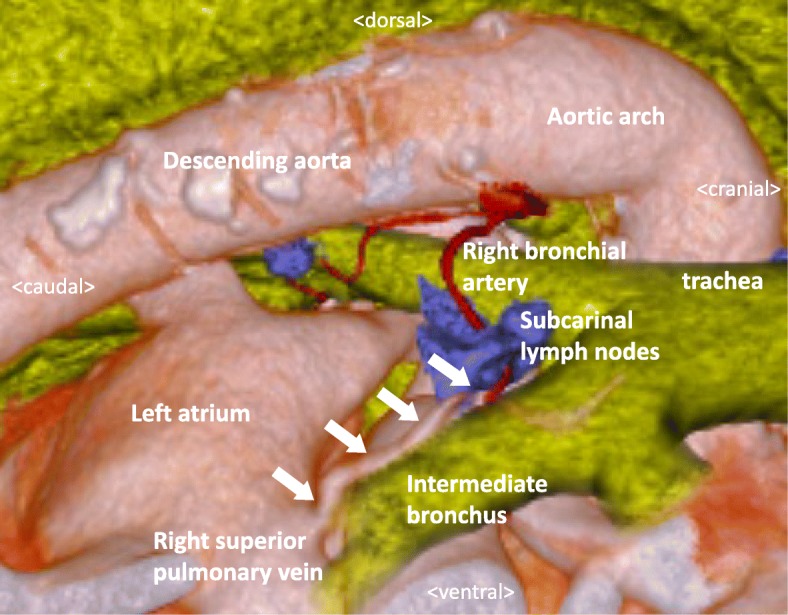
Virtual thoracoscopic image. The anomalous V2 runs behind the intermediate bronchus (white arrows)

**Fig. 4:**
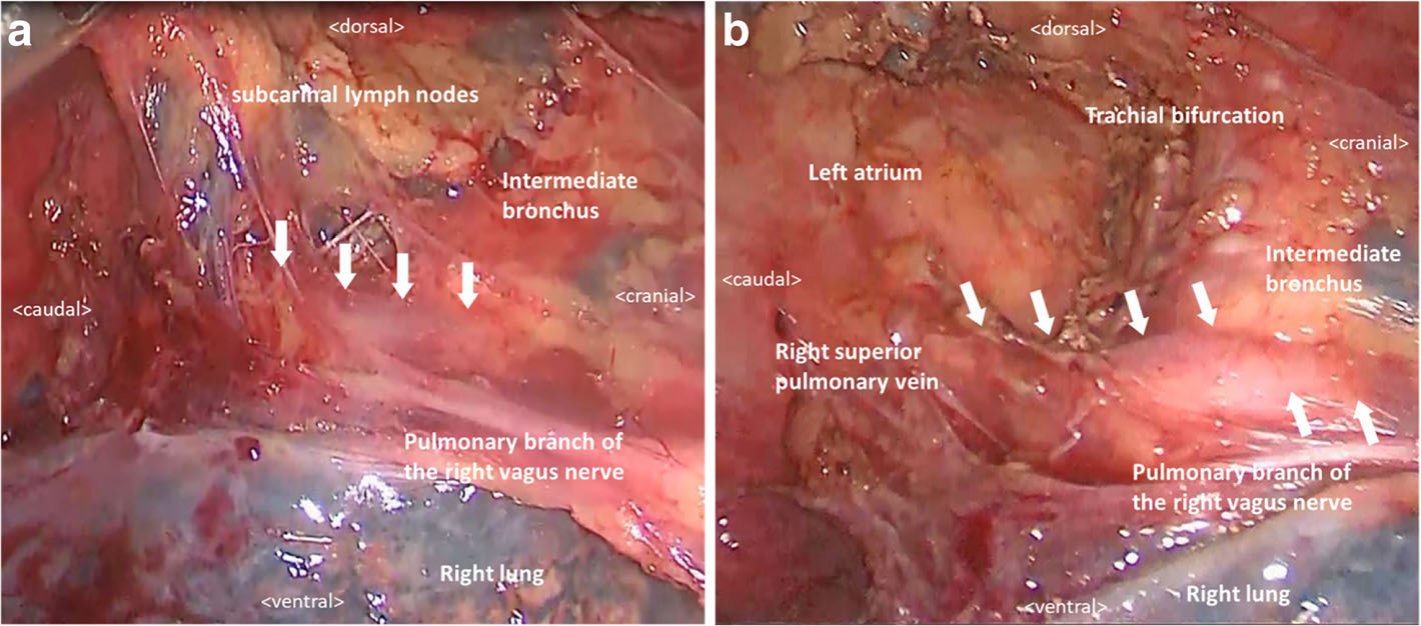
Intraoperative findings. **a** The anomalous V2 identified during the subcarinal lymph node dissection (white arrow). The right bronchus is rolled by pulling the pulmonary branch of the right vagus nerve toward the right side. **b** The anomalous V2 preserved after the dissection (white arrow)

A 61-year-old man with lower thoracic esophageal cancer was referred to our clinic to undergo treatment. His medical history included diabetes mellitus and cataracts. Upper gastrointestinal endoscopy showed a type 3 tumor in the lower thoracic esophagus, and endoscopic biopsy specimen revealed an adenocarcinoma. CT imaging revealed wall thickening in the lower esophagus and swelling of multiple lymph nodes in the mediastinum and abdomen (Fig. 1). His clinical diagnosis based on TNM staging (TNM classification, eighth edition) was cT3 N2 M0 stage IIIB; thus, we selected neoadjuvant chemoradiotherapy (NACRT) as preoperative treatment. Two cycles of 5-fluorouracil (1000 mg/m^2^ from days 1 to 4, and from 29 to 32) and cisplatin (100 mg/m^2^ on days 1 and 29) were administrated intravenously. A total dose of 41.4 Gy was administered in 23 fractions of 1.8 Gy, 5 fractions per week, starting on the first day of the first cycle of chemotherapy. The irradiation field included the primary tumor and the regional lymph nodes, including the subclavian, paraesophageal, subcarinal, and celiac axis lymph nodes. Surgery was performed 5 weeks after the end of the irradiation. During the preoperative examination, contrast-enhanced CT imaging revealed that the anomalous V2 drained into the RSPV, which ran behind the intermediate bronchus (Fig. 2a–d). We reconstructed the CT image using virtual thoracoscopic imaging, and the anomalous V2 was visualized clearly as in the operative view (Fig. 3) using the Ziostation2 workstation (Ziosoft, Inc., Tokyo, Japan). General anesthesia was administered with single-lumen endotracheal intubation for bilateral lung ventilation. Thoracoscopic esophagectomy with lymph node dissection via the right thoracic approach was performed in the prone position under 6–10 mmHg of artificial pneumothorax. Thoracic esophagectomy and mediastinal lymph node dissection were performed using five ports. Subcarinal lymph node dissection started from dissection of the pericardial membrane. Then, the right bronchus was rolled by pulling the right vagus nerve toward the right side and dissecting the subcarinal lymph node from the right bronchus. The anomalous V2 was identified during the lymph node dissection and was preserved without injury (Fig. 4a, b). No visible effects of the radiation, such as fibrosis or edema around the bifurcation, were observed. After the thoracoscopic procedure, we performed hernioplasty for the right inguinal hernia and reconstructed the gastric tube via the posterior mediastinal route in the supine position. The total operation time was 815 min, and the total amount of intraoperative bleeding was 52 g. No severe postoperative complications occurred, except for paralysis of the left recurrent laryngeal nerve. The patient started oral intake on postoperative day (POD) 7 and was discharged on POD 17. The pathological diagnosis on TNM staging was pT3 pN3 cM0 pStage IVA. There were no subcarinal lymph node metastases.

## Conclusions

The branch of the RSPV generally runs inside the right lung. Thus, this vein usually does not appear behind the right main or intermediate bronchus. Therefore, pulmonary vein anomaly carries the risk of massive bleeding during thoracic surgery if not recognized through preoperative examination or intraoperative findings. Akiba et al. reviewed cases of RSPV anomaly and showed that the occurrence rate of the anomaly running behind the intermediate bronchus was 0.3 to 9.3% [4]. After preoperative examination for lung cancer, Shiina et al. reported that anomalous V2 was found in 14 (7.4%) of 189 patients on three-dimensional CT angiography (3-D CTA) [6].

Three reports have described esophagectomy and subcarinal lymph node dissection for patients with esophageal cancer who had a RSPV anomaly (Table 1). Matsubara reported that they could preserve the anomalous RSPV during esophagectomy with thoracotomy for thoracic esophageal cancer [9]. Fujiwara et al. also performed thoracoscopic esophagectomy while preserving the anomalous V2, which drained into the left atrium directly [8]. Shiozaki et al. reported successful subcarinal lymph node dissection using the laparoscopic transhiatal approach for thoracic esophageal cancer with an anomalous V2 [7]. The anomalous pulmonary vein could be preserved during subcarinal lymph node dissection in all the cases but was not detected before the operation.

**Table 1:** Review of previous literature about esophagectomy with a RSPV anomaly

Year	Author	Age	Sex	Origin of anomaly of RSPV	Preoperative recognition (Yes/No)	Operative method	Preservation (Yes/No)
2003	Matsubara	57	M	Right upper lobe	No	Thoracotomy	Yes
2015	Fujiwara et al.	51	M	S2	No	Thoracoscopy (left decubitus)	Yes
2016	Shiozaki et al.	74	M	right upper lobe	No	Laparoscopy (transhiatal)	Yes
	Our case	61	M	S2	Yes	Thoracoscopy (prone)	Yes

*RSPV* right superior pulmonary vein, *S2* posterior segment of the right upper lobe

Recent CT equipment can detect small blood vessels due to improved accuracy of the equipment and the imaging process. Esophageal surgeons mainly use 3-D CTA to check the course of bronchial arteries [10–12]. Our institution has also introduced 3-D CTA for preoperative simulation since 2007, and we have been reconstructing preoperative CT images into virtual thoracoscopic images since then [13]. Virtual imaging has the advantage of being able to provide images with the same view as that in thoracoscopic operation.

Although detection of anomalous V2 might be possible by using enhanced axial CT images [5], without 3-D reconstruction imaging, it is difficult to imagine the locational relationship between this vein and the subcarinal lymph node, right bronchus, and bronchial artery. Our case demonstrates that the right bronchial artery was adjacent to the anomalous V2 (Fig. 3). In the operative view, the anomalous V2 looked like the subcarinal lymph node, which needed to be dissected (Fig. 4a). If we dissected the material from the right bronchus, it would have been easy to recognize the complication. Thus, the exact location of the anomalous V2 should be recognized before and during surgery, and virtual thoracoscopic imaging is useful for identifying its anatomical location.

This is the first report of the application of virtual thoracoscopic imaging for a case with an anatomical anomaly during esophageal cancer surgery. Preoperative diagnosis and virtual thoracoscopic imaging of anomalous V2 can reduce the risk of severe complications during subcarinal lymph node dissection in esophagectomy. More studies are needed to verify the usefulness of virtual thoracoscopic imaging for such cases with anatomical anomalies.

## Abbreviations

3-D CTA: Three-dimensional computed tomographic angiography
Anomalous V2: Anomaly of the superior posterior pulmonary vein in the right upper lobe
CT: Computed tomography
NACRT: Neoadjuvant chemoradiotherapy
POD: Postoperative day
RSPV: Right superior pulmonary vein
